# Supplementary material for: Computational modeling of immersed non-spherical bodies in viscous flows to study embolus-hemodynamics interactions in large-vessel occlusion stroke
Source: Eng Comput. 2026 Jun 29;42(4):134. doi: 10.1007/s00366-026-02364-8 (PMC13315089; doi:10.1007/s00366-026-02364-8)
Supplement: Supplementary file 3 — (pdf 182 KB) [file 366_2026_2364_MOESM3_ESM.pdf]

Computational Modeling Of Embolus Shape Effects In Embolus Hemodynamics Interactions For Stroke

Chayut Teeraratkul, Adarsh Krishnamurthy, Debanjan Mukherjee

Supplementary Material Information

S1 Description of the overall simulation algorithm

Algorithm 1 Fictitious domain IFEM

$\mathcal{M} \leftarrow$ mesh for background fluid domain Ω
$SDF = SDF(\partial\Omega) \leftarrow$ signed distance field for surface mesh of $\mathcal{M}$
$T \leftarrow$ total computational time
$\Delta t \leftarrow$ time-step size
$\underline{\boldsymbol{u}}(t) \leftarrow \underline{\boldsymbol{u}}(t = t0)$ ▷ initial conditions/state
$p(t) \leftarrow p(t = t0)$ ▷ initial conditions/state
while $t < T$ **do**
  $\underline{\boldsymbol{u}}(t) \leftarrow$ the fluid velocity from the previous time step
  $p(t) \leftarrow$ the fluid pressure field from the previous time step
  compute: $\underline{\boldsymbol{F}}^{FSI}$ using $\underline{\boldsymbol{u}}(t)$ and $\underline{\boldsymbol{p}}(t)$ ▷ using Equation 27
  compute: $\underline{\boldsymbol{\tau}}^{FSI}$ using $\underline{\boldsymbol{u}}(t)$ and $\underline{\boldsymbol{p}}(t)$ ▷ using Equation 28
  project: SDF onto solid boundary $\Gamma_s = \partial\Omega_s$
  compute: $\delta(\underline{\boldsymbol{x}})$ contact penetration distance (nodally) ▷ using Equation 48
  compute: $\underline{\boldsymbol{F}}^{COL}$ from $\delta(\underline{\boldsymbol{x}})$ ▷ using Equation 44
  compute: $\underline{\boldsymbol{\tau}}^{COL}$ from $\delta(\underline{\boldsymbol{x}})$ ▷ using Equation 46
  compute: $\underline{\boldsymbol{F}}_{FSI}$ and $\underline{\boldsymbol{\tau}}_{FSI}$ ▷ using Equations 35- 43
  update: particle position $\underline{\boldsymbol{x}}_c$ and velocity $\underline{\boldsymbol{u}}_c$ ▷ using Equations 25
  update: particle orientation $\underline{\boldsymbol{\theta}}_c$ and angular velocity $\underline{\boldsymbol{\omega}}_c$ ▷ using Equations 26
  $\underline{\boldsymbol{u}}(t) \leftarrow$ updated fluid velocity at current time-step ▷ using Equations 7
  $p(t) \leftarrow$ updated fluid pressure at current time-step ▷ using Equations 7
end while

S2 Numerical details of Gear's algorithm

Gear's algorithm consists of two steps: first, for a known position $\underline{\boldsymbol{x}}$ and the corresponding time derivatives $\dot{\underline{\boldsymbol{x}}}, \ddot{\underline{\boldsymbol{x}}}, \dddot{\underline{\boldsymbol{x}}}, \ldots$ at time t, the predictor step estimate the particle's position its time derivative at time $t + \Delta t$ based on Taylor expansion. The degree of truncation depends on the order of integration algorithm. In this work, we employ the fifth order integration algorithm typically employed in DEM and molecular dynamics [38] defined as:

$$\begin{aligned}
\underline{\boldsymbol{x}}^{pr}(t+\Delta t) &= \underline{\boldsymbol{x}}(t) + \Delta t\dot{\underline{\boldsymbol{x}}}(t) + \frac{1}{2}\Delta t^2\ddot{\underline{\boldsymbol{x}}}(t) + \frac{1}{6}\Delta t^3\dddot{\underline{\boldsymbol{x}}}(t) + \frac{1}{24}\Delta t^4\ddddot{\underline{\boldsymbol{x}}}(t) \\
\dot{\underline{\boldsymbol{x}}}^{pr}(t+\Delta t) &= \dot{\underline{\boldsymbol{x}}}(t) + \Delta t\ddot{\underline{\boldsymbol{x}}}(t) + \frac{1}{2}\Delta t^2\dddot{\underline{\boldsymbol{x}}}(t) + \frac{1}{6}\Delta t^3\ddddot{\underline{\boldsymbol{x}}}(t) \\
\ddot{\underline{\boldsymbol{x}}}^{pr}(t+\Delta t) &= \ddot{\underline{\boldsymbol{x}}}(t) + \Delta t\dddot{\underline{\boldsymbol{x}}}(t) + \frac{1}{2}\Delta t^2\ddddot{\underline{\boldsymbol{x}}}(t) \\
\dddot{\underline{\boldsymbol{x}}}^{pr}(t+\Delta t) &= \dddot{\underline{\boldsymbol{x}}}(t) + \Delta t\ddddot{\underline{\boldsymbol{x}}}(t) \\
\ddddot{\underline{\boldsymbol{x}}}^{pr}(t+\Delta t) &= \ddddot{\underline{\boldsymbol{x}}}(t)
\end{aligned} \tag{S1}$$

The acceleration due to the applied force and torque terms are computed at $t+\Delta t$ at the start of integration. The computed accelerations at time $t+\Delta t$, however, usually differ from the predicted value $\ddot{\underline{\boldsymbol{x}}}^{pr}(t+\Delta t)$. Gear's algorithm uses the difference between the predicted and corrected acceleration to compute the correction of the velocity and position. Hence, the second step of Gears integration is the correction step as follows: let the difference between the predicted and corrected acceleration be $\Delta\ddot{\underline{\boldsymbol{x}}} \equiv \ddot{\underline{\boldsymbol{x}}}(t+\Delta t) - \ddot{\underline{\boldsymbol{x}}}^{pr}(t+\Delta t)$. The corrections for the position and the corresponding time derivatives are computed as follows

$$
\begin{aligned}
\underline{\boldsymbol{x}}(t+\Delta t) &= \underline{\boldsymbol{x}}^{pr}(t+\Delta t) + c_0\left(\frac{\Delta t^2}{2}\Delta\ddot{\underline{\boldsymbol{x}}}\right) \\
\dot{\underline{\boldsymbol{x}}}(t+\Delta t) &= \dot{\underline{\boldsymbol{x}}}^{pr}(t+\Delta t) + c_1\frac{1}{\Delta t}\left(\frac{\Delta t^2}{2}\Delta\ddot{\underline{\boldsymbol{x}}}\right) \\
\ddot{\underline{\boldsymbol{x}}}(t+\Delta t) &= \ddot{\underline{\boldsymbol{x}}}^{pr}(t+\Delta t) + c_2\frac{2}{\Delta t^2}\left(\frac{\Delta t^2}{2}\Delta\ddot{\underline{\boldsymbol{x}}}\right) \\
\dddot{\underline{\boldsymbol{x}}}(t+\Delta t) &= \dddot{\underline{\boldsymbol{x}}}^{pr}(t+\Delta t) + c_3\frac{6}{\Delta t^3}\left(\frac{\Delta t^2}{2}\Delta\ddot{\underline{\boldsymbol{x}}}\right) \\
\ddddot{\underline{\boldsymbol{x}}}(t+\Delta t) &= \ddddot{\underline{\boldsymbol{x}}}^{pr}(t+\Delta t) + c_4\frac{24}{\Delta t^3}\left(\frac{\Delta t^3}{2}\Delta\ddot{\underline{\boldsymbol{x}}}\right)
\end{aligned}
\tag{S2}
$$

With the coefficients of fifth order Gears integration are as follows

$$
c_0 = \frac{19}{90}\,, c_1 = \frac{3}{4}\,, c_2 = 1\,, c_3 = \frac{1}{2}\,, c_4 = \frac{1}{12} \tag{S3}
$$

S3 Additional animations for illustration of embolus dynamics

We have included here two additional animation files that represent the dynamics of large occlusive emboli of spherical and non-spherical geometries moving within an anatomically realistic segment of the human carotid bifurcation. The animation titled `anatomical-E-combo.mp4` demonstrates the dynamics of ellipsoidal shaped emboli of two different sizes ; while the animaton title `anatomical-S-combo.mp4` demonstrates the dynamics of spherical emboli of two different sizes. The animation clearly shows the differences in tumble and rotation dynamics as experience by large non-spherical emboli, as they interact with the fluid flow patterns and the curved vessel walls.
